# Association of Prior SARS-CoV-2 Infection with Immune Activation in Virally Suppressed People Living with HIV

**DOI:** 10.3390/microorganisms14081624

**Published:** 2026-07-24

**Authors:** Madalina-Ianca Suba, Ovidiu Rosca, Bogdan Hogea, Camelia Corina Pescaru, Florina Cristiana Lucaciu, Ahmed Abu-Awwad, Adrian-Cosmin Ilie, Daniel Pop, Simona-Alina Abu-Awwad

**Affiliations:** 1 Methodological and Infectious Diseases Research Center, Faculty of Medicine, “Victor Babes” University of Medicine and Pharmacy, 300041 Timisoara, Romania; madalina.suba@umft.ro (M.-I.S.); ovidiu.rosca@umft.ro (O.R.); 2 Clinical Hospital of Infectious Diseases and Pulmonology “Victor Babes”, Gheorghe Adam Street 13, 300310 Timisoara, Romania; florina.lucaciu@umft.ro; 3 “Pius Brinzeu” Emergency Clinical County Hospital, Bld Liviu Rebreanu, No. 156, 300723 Timisoara, Romania; alina.abuawwad@umft.ro; 4 Department XV, Discipline of Orthopedics-Traumatology, “Victor Babes” University of Medicine and Pharmacy, Eftimie Murgu Square, No. 2, 300041 Timisoara, Romania; daniel.pop@umft.ro; 5 Profesor Universitar Doctor Teodor Șora Research Centre, “Victor Babes” University of Medicine and Pharmacy, Eftimie Murgu Square, No. 2, 300041 Timisoara, Romania; 6 Center for Research and Innovation in Personalized Medicine of Respiratory Diseases (CRIPMRD), “Victor Babes” University of Medicine and Pharmacy, Eftimie Murgu Square 2, 300041 Timisoara, Romania; pescaru.camelia@umft.ro; 7 Doctoral School, “Victor Babes” University of Medicine and Pharmacy, 2 Eftimie Murgu Square, 300041 Timisoara, Romania; 8 Department of Public Health and Sanitary Management, “Victor Babes” University of Medicine and Pharmacy, Eftimie Murgu Square 2, 300041 Timisoara, Romania; ilie.adrian@umft.ro; 9 Centre for Translational Research and Systems Medicine, Faculty of Medicine, “Victor Babes” University of Medicine and Pharmacy, Eftimie Murgu Sq. No. 2, 300041 Timisoara, Romania; 10 Department of Obstetrics and Gynecology, “Victor Babes” University of Medicine and Pharmacy, 300041 Timisoara, Romania

**Keywords:** HIV, SARS-CoV-2, immune activation, chronic inflammation, antiretroviral therapy, viral suppression, IL-6, C-reactive protein, metabolic syndrome, long COVID

## Abstract

Residual inflammation remains a hallmark of treated HIV infection despite durable viral suppression. Whether previous SARS-CoV-2 infection is associated with residual inflammation in people living with HIV (PLWH) remains incompletely understood. This study evaluated the association between previous COVID-19 and persistent inflammation in virally suppressed PLWH. In this retrospective observational single-center study, 286 adults receiving antiretroviral therapy between January 2023 and December 2025 were included. Patients were stratified according to documented SARS-CoV-2 infection history. A secondary analysis included 231 individuals with sustained viral suppression (HIV-RNA < 50 copies/mL). Inflammatory biomarkers, immune recovery parameters, metabolic characteristics, and independent predictors of elevated inflammatory biomarker levels were evaluated. Previous SARS-CoV-2 infection was associated with significantly higher concentrations of C-reactive protein, interleukin-6, tumor necrosis factor-α, erythrocyte sedimentation rate, neutrophil-to-lymphocyte ratio, and platelet-to-lymphocyte ratio (all *p* < 0.01). Among virally suppressed patients, elevated inflammatory biomarker levels were associated with lower CD4+ T-cell counts, lower CD4/CD8 ratios, obesity, metabolic syndrome, dyslipidemia, and hepatic steatosis. Previous SARS-CoV-2 infection, obesity, metabolic syndrome, and CD4+ T-cell counts < 500 cells/mm^3^ were independently associated with elevated inflammatory biomarker levels. Previous SARS-CoV-2 infection was independently associated with an unfavorable inflammatory profile in virally suppressed people living with HIV. Given the retrospective observational design, these findings should be interpreted as associations rather than evidence of causality. These findings suggest that long-term host-related and metabolic factors may contribute to residual inflammation beyond viral control and highlight the need for prospective studies investigating strategies to reduce residual immune activation in treated HIV infection.

## 1. Introduction

The widespread implementation of combination antiretroviral therapy (ART) has profoundly changed the natural history of HIV infection [1]. For most people living with HIV, durable viral suppression is now an achievable therapeutic goal, transforming what was once a fatal disease into a chronic condition with a near-normal life expectancy [2]. Nevertheless, the absence of detectable plasma HIV-RNA does not necessarily indicate complete immunological recovery. A considerable proportion of treated individuals continue to exhibit evidence of residual inflammation despite years of successful virological control, suggesting that mechanisms other than active viral replication contribute to chronic inflammation [3].

Current evidence indicates that residual immune activation results from a complex interplay of biological processes rather than a single pathogenic mechanism [4,5]. Persistent viral reservoirs, continuous antigenic stimulation, microbial translocation secondary to intestinal barrier dysfunction, and long-lasting activation of innate immune cells have all been implicated in maintaining a chronic inflammatory milieu during treated HIV infection [6,7]. These processes promote sustained production of inflammatory mediators, including interleukin-6 (IL-6), tumor necrosis factor-alpha (TNF-α), and C-reactive protein (CRP), biomarkers that have repeatedly been associated with cardiovascular disease, metabolic dysfunction, immune senescence, and increased long-term mortality among people receiving suppressive ART [8].

The COVID-19 pandemic introduced an additional challenge for this already dysregulated immune environment. Although SARS-CoV-2 is primarily regarded as an acute respiratory pathogen, it has become increasingly evident that its biological effects frequently extend far beyond the resolution of the initial infection [9,10,11]. Persistent cytokine production, prolonged monocyte activation, endothelial dysfunction, and altered T-cell responses have all been described months after viral clearance [12]. Interestingly, many of these pathways overlap with the mechanisms already known to sustain chronic inflammation in HIV infection [13,14,15].

This overlap raises an important biological question. Could previous SARS-CoV-2 infection amplify the residual immune activation already present in people living with HIV, even after both viruses are effectively controlled? From an immunological perspective, such an interaction appears plausible [16,17]. HIV establishes long-lived viral reservoirs that continuously stimulate the immune system despite suppressive ART, whereas SARS-CoV-2 may leave behind a prolonged inflammatory imprint characterized by persistent activation of innate and adaptive immune responses. Together, these mechanisms may reinforce one another, contributing to a state of chronic immune dysregulation that extends well beyond the acute phases of either infection [18,19].

Despite growing interest in long COVID and chronic immune activation, relatively little is known about the inflammatory profile of virally suppressed people living with HIV following SARS-CoV-2 infection [20,21].

Most available studies have focused either on acute COVID-19 outcomes or on HIV-associated inflammation independently, while the interaction between these two conditions remains insufficiently characterized [22,23]. Clarifying this relationship is clinically relevant because residual immune activation is associated with non-AIDS comorbidities and may influence long-term prognosis even in patients with excellent virological control. Although previous studies have described chronic immune activation in treated HIV infection and post-COVID-19 inflammatory alterations separately, few have specifically investigated the inflammatory profile of virally suppressed people living with HIV with documented prior SARS-CoV-2 infection in routine clinical practice. Furthermore, limited data are available regarding the combined associations of previous COVID-19, immune recovery, and metabolic comorbidities with residual inflammation in this population. By simultaneously evaluating inflammatory biomarkers, immune recovery parameters, metabolic characteristics, and independent predictors of residual inflammation, the present study provides additional real-world evidence addressing these knowledge gaps [24,25].

Based on the overlapping mechanisms of immune dysregulation in treated HIV infection and following SARS-CoV-2 infection, we hypothesized that virally suppressed people living with HIV with a documented history of COVID-19 would exhibit a less favorable inflammatory profile than those without documented SARS-CoV-2 infection. Therefore, we investigated inflammatory activity in a real-world cohort of people living with HIV receiving antiretroviral therapy during the post-COVID-19 period. Specifically, we evaluated whether previous SARS-CoV-2 infection was associated with higher inflammatory biomarker levels and explored the relationships between inflammatory status, immune recovery, metabolic abnormalities, and virological suppression.

## 2. Materials and Methods

### 2.1. Study Design and Population

This retrospective observational study was conducted at the “Victor Babeș” Clinical Hospital for Infectious Diseases and Pneumophthisiology, Timișoara, Romania. The study analyzed HIV-positive patients monitored during a three-year period, between January 2023 and December 2025, corresponding to the post-COVID-19 era, when routine HIV care and long-term follow-up activities had been fully re-established following the major pandemic waves.

The primary objective of the study was to evaluate the persistence of systemic inflammation among people living with HIV in relation to previous SARS-CoV-2 infection and virological suppression status. Particular emphasis was placed on patients with sustained viral suppression, as residual immune activation has increasingly been recognized as a clinically relevant phenomenon despite successful antiretroviral therapy.

**Figure 1:**
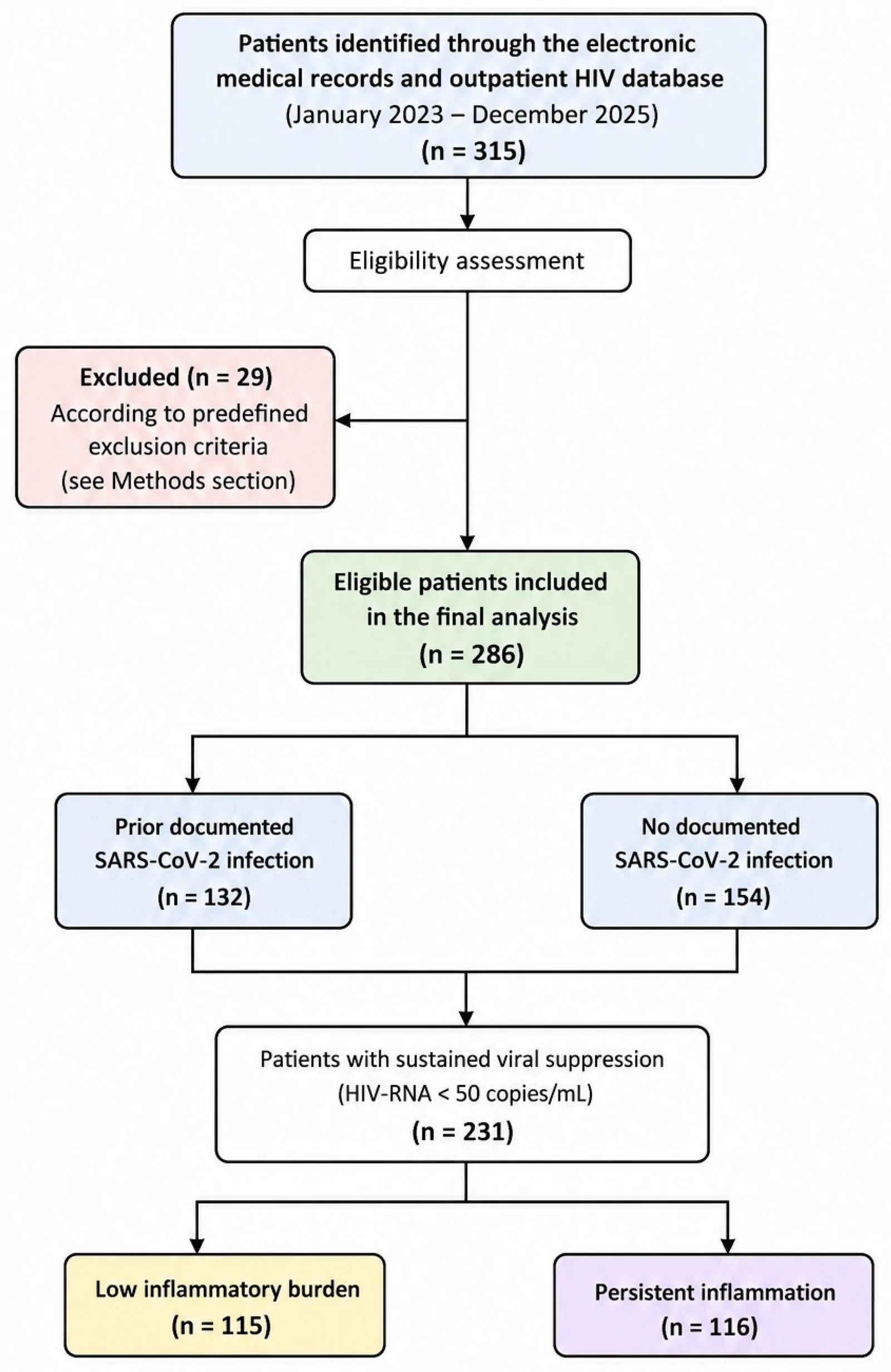
Flow diagram illustrating patient identification, eligibility assessment, application of the predefined exclusion criteria, inclusion in the final study cohort, allocation according to documented SARS-CoV-2 infection history, and selection of the virally suppressed subgroup for the secondary analysis.

All potentially eligible patients were identified through the hospital’s electronic medical records and outpatient HIV database. Consecutive sampling was used, and all patients who fulfilled the predefined inclusion and exclusion criteria during the study period were included in the analysis. No additional selection or sampling procedure was applied. All potentially eligible patients were identified consecutively through the hospital’s electronic medical records and outpatient HIV database. During the study period, 315 patients were screened for eligibility. After application of the predefined inclusion and exclusion criteria, 286 patients fulfilled all eligibility criteria and were included in the final analysis. No additional sampling or selection procedure was applied. A flow diagram of patient selection is presented in Figure 1.

During the preparation of this manuscript, the authors used Grammarly for proofreading and language editing. The scientific content of the manuscript was entirely written by the authors. The authors reviewed and edited the output where applicable and take full responsibility for the content of this publication.

#### 2.1.1. Inclusion Criteria

Patients were considered eligible if they met all of the following criteria:

- Age ≥ 18 years.
- Confirmed HIV-1 infection based on national diagnostic criteria.
- Active follow-up at our institution during the study period.
- Receipt of antiretroviral therapy for at least 12 consecutive months prior to evaluation.
- Availability of at least one documented HIV viral load determination during the study period.
- Availability of immunological assessment, including CD4+ T-cell count and CD4/CD8 ratio.
- Availability of inflammatory biomarker measurements, including CRP, IL-6, and TNF-α.
- Availability of complete demographic, clinical, and treatment-related information in the medical records.
- Documented information regarding previous SARS-CoV-2 infection status.

#### 2.1.2. Exclusion Criteria

Patients were excluded if any of the following conditions were present:

- Newly diagnosed HIV infection (<12 months from diagnosis).
- Documented interruption of antiretroviral therapy exceeding 30 consecutive days during the study period.
- Acute bacterial, viral, fungal, or parasitic infection at the time of laboratory assessment.
- Hospitalization for any acute infectious condition within the previous four weeks.
- Active opportunistic infection, including tuberculosis, pneumocystosis, cryptococcosis, cytomegalovirus disease, or other AIDS-defining infections.
- Active malignant disease or ongoing chemotherapy.
- Known autoimmune or systemic inflammatory disorders (e.g., rheumatoid arthritis, systemic lupus erythematosus, inflammatory bowel disease, vasculitis).
- Chronic immunosuppressive therapy, including systemic corticosteroids, biologic agents, or cytotoxic drugs.
- Decompensated liver disease, acute hepatitis, or acute liver failure.
- End-stage renal disease requiring dialysis.
- Pregnancy or postpartum status.
- Active substance abuse likely to interfere with treatment adherence or follow-up.
- Incomplete clinical records.
- Duplicate records or repeated evaluations from the same patient.

After application of the inclusion and exclusion criteria, 286 patients were considered eligible and constituted the final study cohort.

### 2.2. Group Allocation

For the primary analysis, patients were stratified according to their documented history of SARS-CoV-2 infection during the study period. The objective of this comparison was to investigate whether previous COVID-19 was associated with an unfavorable inflammatory profile among people living with HIV, despite contemporary antiretroviral treatment and generally favorable virological outcomes.

Based on the available medical documentation, patients were allocated to one of two groups:

Prior COVID-19 group, consisting of patients with documented evidence of previous SARS-CoV-2 infection.

No documented COVID-19 group, consisting of patients without evidence of previous SARS-CoV-2 infection in their medical records.

A previous SARS-CoV-2 infection was considered present when at least one of the following criteria was fulfilled: a positive reverse-transcription polymerase chain reaction (RT-PCR) test, a positive rapid antigen test documented in the medical record, hospital discharge documentation related to COVID-19, or a clearly documented diagnosis established by an infectious diseases specialist. Information regarding previous COVID-19 episodes was retrieved from hospital records, outpatient follow-up documentation, and available electronic medical databases.

Patients without documented laboratory confirmation or medical documentation suggestive of previous SARS-CoV-2 infection were classified as having no documented COVID-19 history. Because asymptomatic or untested infections may have occurred during the pandemic, this group was designated as “no documented COVID-19” rather than “COVID-19 negative.”

To further explore the relationship between virological control and residual immune activation, a secondary analysis was performed exclusively among patients who achieved sustained viral suppression. Viral suppression was defined as an HIV-RNA level below 50 copies/mL [26] at the time of evaluation, consistent with current international treatment targets and routine clinical practice.

Within this virally suppressed subgroup, patients were subsequently categorized as having either low inflammatory burden or persistent inflammation according to the predefined criteria described in Section 2.3. This approach allowed us to identify patients who continued to exhibit evidence of systemic immune activation despite successful suppression of viral replication.

The rationale for this secondary stratification was that persistent inflammation has emerged as one of the principal mechanisms underlying non-AIDS comorbidities in people living with HIV. Therefore, examining differences between virally suppressed patients with and without evidence of ongoing inflammation may provide clinically relevant insights regarding the long-term consequences of HIV infection in the post-COVID-19 era.

### 2.3. Definition of Inflammatory Status

Patients were classified as having persistent inflammation if they fulfilled at least one of the following predefined criteria: serum C-reactive protein (CRP) > 10 mg/L or an interleukin-6 (IL-6) concentration above the cohort median. CRP was selected because it is a widely validated marker of systemic inflammation and has been extensively used in studies evaluating chronic immune activation in people living with HIV. IL-6 was included because it represents a key cytokine involved in residual inflammation and has consistently been associated with residual inflammation and adverse long-term outcomes in treated HIV infection [24,27].

Because no universally accepted clinical threshold exists for defining residual inflammation based on IL-6 concentrations in virally suppressed people living with HIV, the cohort median was selected as a pragmatic distribution-based cut-off to distinguish patients with relatively higher inflammatory activity. Similar distribution-based approaches have frequently been adopted in observational biomarker studies when validated clinical thresholds are unavailable [28,29]. This strategy was intended to facilitate comparisons within the study population rather than to establish a clinically validated diagnostic threshold for chronic inflammation. Patients who did not meet either criterion were classified as having low inflammatory burden.

Additional inflammatory parameters, including tumor necrosis factor-α (TNF-α), erythrocyte sedimentation rate (ESR), neutrophil-to-lymphocyte ratio (NLR), and platelet-to-lymphocyte ratio (PLR), were analyzed to provide a more comprehensive characterization of the inflammatory profile. However, these biomarkers were not incorporated into the primary definition of persistent inflammation because they were evaluated as secondary inflammatory markers rather than as classification criteria.

To minimize the influence of transient inflammatory responses, patients with acute infections, active opportunistic diseases, autoimmune disorders, malignancies, or other clinically evident causes of systemic inflammation were excluded from the analysis. Consequently, elevated inflammatory biomarker levels were considered more likely to reflect chronic residual immune activation than an acute inflammatory process.

### 2.4. Data Collection and Laboratory Assessment

Clinical, demographic, laboratory, and treatment-related data were collected retrospectively from electronic medical records, outpatient follow-up documentation, laboratory reports, and institutional databases. For patients with multiple evaluations during the study period, the most recent complete assessment meeting all eligibility criteria was included in the analysis.

The following demographic and clinical variables were recorded: age, sex, body mass index (BMI), smoking status, duration of HIV infection, duration of antiretroviral therapy (ART), history of SARS-CoV-2 infection, and current antiretroviral treatment regimen.

Virological and immunological data included HIV-RNA viral load, CD4+ T-cell count, and CD4/CD8 ratio. Viral suppression was defined as an HIV-RNA level below 50 copies/mL at the time of evaluation.

Inflammatory parameters collected for analysis included C-reactive protein (CRP), interleukin-6 (IL-6), tumor necrosis factor-alpha (TNF-α), erythrocyte sedimentation rate (ESR), total leukocyte count, neutrophil percentage, lymphocyte percentage, neutrophil-to-lymphocyte ratio (NLR), and platelet-to-lymphocyte ratio (PLR).

Information regarding HIV-related and non-HIV-related comorbidities was also extracted from medical records. Particular attention was given to metabolic and hepatic conditions, including obesity, metabolic syndrome, dyslipidemia, and hepatic steatosis. Metabolic syndrome was defined according to the diagnosis recorded in the patient’s medical file, while dyslipidemia was considered present in patients with a documented diagnosis, ongoing lipid-lowering treatment, or abnormal lipid profile values requiring clinical follow-up. Hepatic steatosis was considered present when documented by abdominal ultrasonography or reported in the patient’s medical records.

Additional clinical information regarding previous COVID-19 infection, smoking status, and treatment history was obtained from routine follow-up visits and hospitalization records. All collected data were anonymized before statistical analysis to ensure patient confidentiality.

All laboratory parameters were obtained as part of routine clinical follow-up and were extracted from the hospital laboratory database. For each patient, the laboratory assessment closest to the study evaluation date was considered for analysis.

The inflammatory profile was evaluated using serum C-reactive protein (CRP), interleukin-6 (IL-6), tumor necrosis factor-alpha (TNF-α), and erythrocyte sedimentation rate (ESR). In addition, several inflammation-related hematological indices were assessed, including total leukocyte count, neutrophil percentage, lymphocyte percentage, neutrophil-to-lymphocyte ratio (NLR), and platelet-to-lymphocyte ratio (PLR).

Immunological status was assessed using CD4+ T-cell count and CD4/CD8 ratio, both of which are routinely used markers of immune recovery and disease monitoring in people living with HIV. Virological control was evaluated using plasma HIV-RNA levels. Sustained viral suppression was defined as an HIV-RNA level below 50 copies/mL, in accordance with current international treatment recommendations and routine clinical practice [30].

Inflammatory biomarkers were analyzed to characterize the degree of residual immune activation and to investigate their relationship with previous SARS-CoV-2 infection, immune recovery, and metabolic comorbidities. Hematological indices derived from complete blood count parameters, particularly NLR and PLR, were included because of their recognized role as accessible markers of systemic inflammation [31].

All laboratory investigations were performed in the accredited clinical laboratory of our institution using standardized analytical methods and quality-control procedures routinely employed in patient care.

### 2.5. Antiretroviral Therapy

Information regarding antiretroviral treatment was collected from the patients’ medical records and outpatient follow-up documentation. For each participant, the current ART regimen, treatment duration, and virological status at the time of evaluation were recorded.

To facilitate the analysis, treatment regimens were categorized according to the anchor antiretroviral agent into bictegravir/tenofovir alafenamide/emtricitabine (BIC/TAF/FTC)-based therapy, dolutegravir-based therapy, protease inhibitor (PI)-based therapy, and other ART combinations. Treatment duration was calculated as the interval between ART initiation and the date of the study evaluation.

Because all patients were managed according to contemporary treatment recommendations, the majority were receiving integrase strand transfer inhibitor (INSTI)-based regimens. However, PI-based and other treatment combinations remained represented, mainly in patients with longer HIV duration, previous treatment modifications, comorbidities, or specific therapeutic considerations [32].

Antiretroviral therapy was included in the analysis because both treatment class and treatment exposure may influence long-term clinical outcomes. Previous studies have suggested potential associations between specific ART regimens and metabolic abnormalities, weight gain, hepatic steatosis, lipid disturbances, and markers of residual inflammation. Therefore, the current treatment regimen was evaluated as a potential factor associated with persistent inflammatory activity in virally suppressed patients [33,34].

In addition, treatment duration was analyzed as a marker of cumulative ART exposure and long-term HIV management, given its possible influence on immune recovery and chronic inflammatory status.

### 2.6. Study Outcomes

The primary outcome of the study was the identification of elevated inflammatory biomarkers among people living with HIV who achieved sustained virological suppression. Specifically, we sought to determine whether evidence of systemic inflammation persisted despite effective antiretroviral therapy and successful control of HIV replication, reflecting long-term immune dysregulation rather than ongoing viral replication.

Secondary outcomes focused on characterizing the inflammatory phenotype associated with previous SARS-CoV-2 infection. To this end, inflammatory biomarker profiles were compared between patients with and without a documented history of COVID-19, and demographic, immunological, metabolic, and HIV-related characteristics associated with increased inflammatory activity were evaluated.

Because residual inflammation has been closely linked to incomplete immune restoration, we further investigated the relationship between inflammatory biomarkers and markers of immune recovery. Particular attention was given to the association between CRP and IL-6 concentrations and established immunological parameters, including CD4+ T-cell count and the CD4/CD8 ratio, in order to explore whether residual inflammation was accompanied by less favorable immune reconstitution despite suppressive ART.

Finally, multivariable logistic regression analysis was performed to identify independent determinants of residual inflammation in virally suppressed individuals. The model evaluated the contribution of previous SARS-CoV-2 infection together with host-related factors, including immune status, metabolic abnormalities, obesity, HIV disease characteristics, and antiretroviral treatment, to better define the biological and clinical factors associated with chronic inflammatory activity.

### 2.7. Statistical Analysis

Statistical analysis was performed using IBM SPSS Statistics for Windows, Version 29.0 (IBM Corp., Armonk, NY, USA). The distribution of continuous variables was assessed using the Shapiro–Wilk test together with visual inspection of histograms and Q–Q plots. Variables showing an approximately normal distribution were analyzed using parametric tests. Continuous variables were expressed as mean ± standard deviation (SD), while categorical variables were presented as absolute frequencies and percentages.

Comparisons between patients with and without documented SARS-CoV-2 infection, as well as between virally suppressed patients with low inflammatory burden and persistent inflammation, were performed using the independent samples *t*-test for continuous variables and the chi-square test for categorical variables. Fisher’s exact test was applied when appropriate.

To investigate the relationship between inflammatory biomarkers and immune status, correlation analyses were performed between CRP, IL-6, and CD4+ T-cell count. Correlation coefficients were calculated, and the results were illustrated using scatter plots with linear regression lines and corresponding 95% confidence intervals.

To identify factors independently associated with persistent inflammation among virally suppressed patients, a multivariable logistic regression analysis was performed. Variables were selected a priori based on their clinical relevance, evidence from the existing literature, and the results of the univariate analyses. To avoid model overfitting, only variables considered clinically relevant and supported by previous evidence, together with those showing potential associations in the univariate analyses, were entered into the multivariable model.

Accordingly, the following variables were entered into the multivariable logistic regression model: age > 45 years, obesity (BMI ≥ 30 kg/m^2^), prior SARS-CoV-2 infection, metabolic syndrome, HIV infection duration > 10 years, CD4+ T-cell count < 500 cells/mm^3^, and treatment with a bictegravir/tenofovir alafenamide/emtricitabine (BIC/TAF/FTC)-based regimen. Model calibration was assessed using the Hosmer–Lemeshow goodness-of-fit test, while overall model performance was evaluated using Nagelkerke’s R^2^. The discriminatory ability of the model was additionally evaluated using the area under the receiver operating characteristic curve (AUC).

The results of the logistic regression analysis were reported as odds ratios (ORs) with corresponding 95% confidence intervals (CIs). Statistical significance was defined as a two-sided *p*-value < 0.05.

Only patients with complete data for all variables included in the analyses were retained. Consequently, no imputation methods for missing data were required.

Multicollinearity among the independent variables included in the multivariable model was assessed using variance inflation factors (VIFs) and tolerance statistics. No evidence of problematic multicollinearity was observed (all VIF values < 2.5). All patients included in the final analyses had complete biomarker data; therefore, no imputation procedures were required.

### 2.8. Ethical Considerations

The study was conducted in accordance with the principles of the Declaration of Helsinki and was approved by the local Ethics Committee (6245/25 April 2026). Because of the retrospective design of the study, all data were anonymized before analysis, and no information allowing patient identification was included in the final database.

## 3. Results

A total of 286 HIV-positive patients met the eligibility criteria and were included in the final analysis. Of these, 132 (46.2%) had a documented history of SARS-CoV-2 infection, while 154 (53.8%) had no documented evidence of previous COVID-19.

### 3.1. Baseline Characteristics According to SARS-CoV-2 Infection History

The baseline characteristics of the study population are presented in Table 1. The two groups were generally comparable with regard to age, sex distribution, smoking status, duration of HIV infection, duration of antiretroviral therapy, virological suppression rates, CD4+ T-cell count, and CD4/CD8 ratio.

Patients with a history of COVID-19 had a significantly higher body mass index compared with those without documented SARS-CoV-2 infection (27.4 ± 5.1 vs. 26.1 ± 4.5 kg/m^2^, *p* = 0.024). In addition, metabolic syndrome was more frequently observed among patients with prior COVID-19 (34.8% vs. 23.4%, *p* = 0.033), as was hepatic steatosis (31.8% vs. 20.8%, *p* = 0.034).

Although dyslipidemia was also more common in the prior COVID-19 group, the difference did not reach statistical significance (46.2% vs. 35.7%, *p* = 0.071). No significant differences were identified regarding the distribution of antiretroviral regimens between the two groups.

Overall, these findings suggest that patients with previous SARS-CoV-2 infection exhibited a less favorable metabolic profile despite similar HIV-related clinical characteristics and comparable rates of virological control.

### 3.2. Inflammatory Biomarkers According to SARS-CoV-2 Infection History

As shown in Table 2, patients with documented prior COVID-19 demonstrated consistently higher levels of inflammatory biomarkers compared with those without documented SARS-CoV-2 infection.

Mean CRP levels were significantly higher in the prior COVID-19 group (5.8 ± 3.4 vs. 3.9 ± 2.7 mg/L, *p* < 0.001). Similar differences were observed for IL-6 (7.4 ± 3.1 vs. 5.1 ± 2.6 pg/mL, *p* < 0.001) and TNF-α (12.6 ± 4.8 vs. 9.8 ± 3.9 pg/mL, *p* < 0.001). Patients with previous COVID-19 also had higher ESR values (21.3 ± 10.4 vs. 16.8 ± 8.7 mm/h, *p* = 0.001).

With respect to hematological inflammatory indices, the prior COVID-19 group exhibited higher neutrophil percentages, lower lymphocyte percentages, and significantly increased NLR and PLR values. In contrast, total leukocyte count did not differ significantly between groups.

Taken together, these findings indicate that previous SARS-CoV-2 infection was associated with a persistently elevated inflammatory profile in people living with HIV, even during routine follow-up in the post-pandemic period.

### 3.3. Characteristics of Virally Suppressed Patients According to Inflammatory Status

Among the 231 patients who achieved viral suppression (HIV-RNA < 50 copies/mL), 115 were classified as having a low inflammatory burden and 116 as having persistent inflammation.

As presented in Table 3, patients with elevated inflammatory biomarkers were significantly older and had a higher body mass index compared with those in the low-inflammation group. Metabolic syndrome, dyslipidemia, and hepatic steatosis were all substantially more frequent among patients with elevated inflammatory biomarkers.

Markers of immune recovery also differed between groups. Patients with elevated inflammatory biomarkers had lower CD4+ T-cell counts and lower CD4/CD8 ratios, suggesting less favorable immunological restoration despite successful suppression of HIV replication.

These results support the concept that residual inflammation remains closely linked to both metabolic abnormalities and incomplete immune recovery, even among patients receiving effective antiretroviral therapy.

### 3.4. Relationship Between Inflammatory Biomarkers and Immune Recovery

To further explore the relationship between residual inflammation and immune recovery, correlation analyses were performed among virally suppressed patients (Figure 1).

A significant inverse correlation was observed between CRP levels and CD4+ T-cell count (r = −0.31, *p* < 0.001), indicating that patients with better immune recovery tended to exhibit lower levels of systemic inflammation. Similarly, IL-6 demonstrated a moderate negative correlation with CD4 count (r = −0.37, *p* < 0.001).

**Figure 2:**
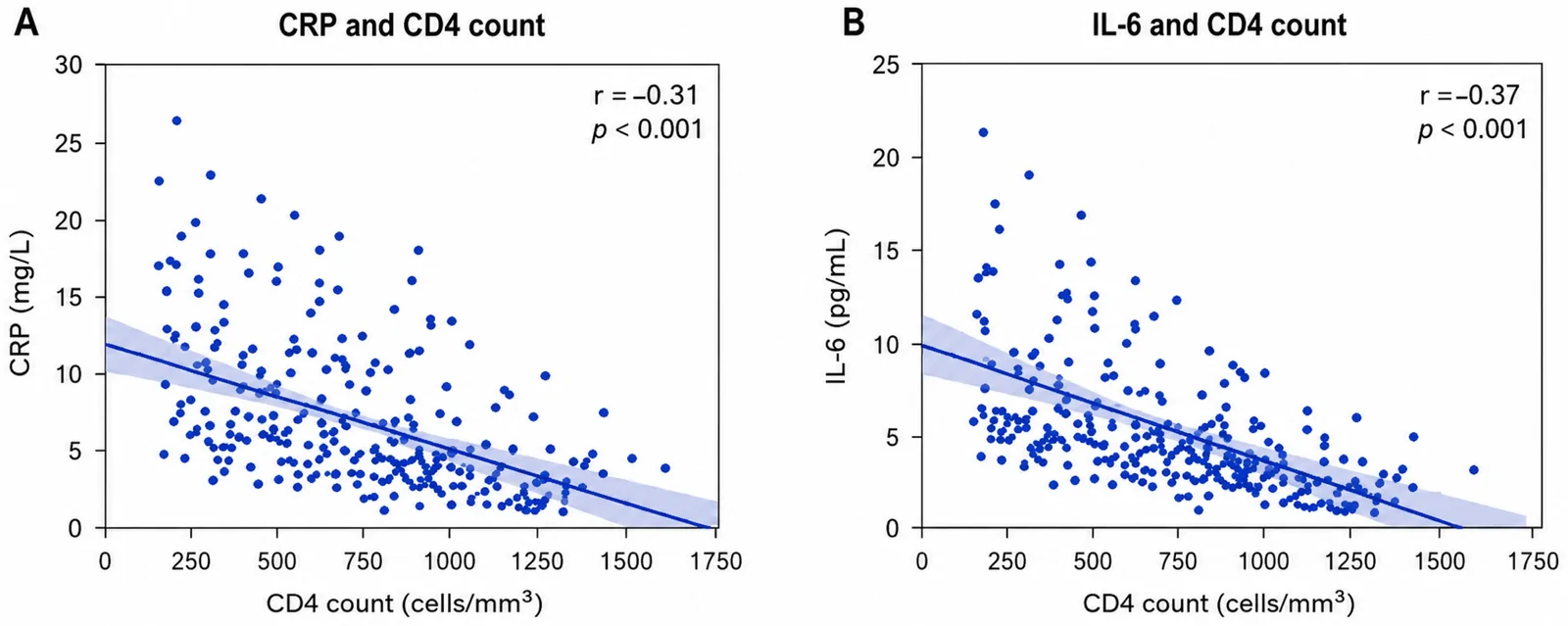
Association between inflammatory biomarkers and immune status in virally suppressed HIV-positive patients (*n* = 231). Scatter plots illustrating the relationship between CD4+ T-cell count and two inflammatory biomarkers among virally suppressed participants. Each point represents one patient. The solid blue line indicates the fitted linear regression, and the shaded area represents the corresponding 95% confidence interval. Pearson correlation coefficients (r) and associated *p*-values are shown in the upper right corner of each panel. (**A**) Relationship between serum C-reactive protein (CRP, mg/L) and CD4+ T-cell count (cells/mm^3^) (r = −0.31, *p* < 0.001). (**B**) Relationship between serum interleukin-6 (IL-6, pg/mL) and CD4+ T-cell count (cells/mm^3^) (r = −0.37, *p* < 0.001). In both analyses, higher CD4+ T-cell counts were associated with lower inflammatory biomarker levels, indicating an inverse relationship between immune recovery and systemic inflammation.

As illustrated in Figure 2, increasing CD4+ T-cell counts were associated with progressively lower concentrations of both CRP and IL-6. These findings suggest that more effective immune reconstitution is accompanied by reduced residual inflammatory activity, even among patients who have already achieved virological suppression.

Overall, higher levels of inflammatory biomarkers were independently associated with less favorable immunological recovery, supporting the concept that residual immune activation may persist despite successful control of HIV replication.

### 3.5. Predictors of Elevated Inflammatory Biomarker Levels in Virally Suppressed Patients

**Figure 3:**
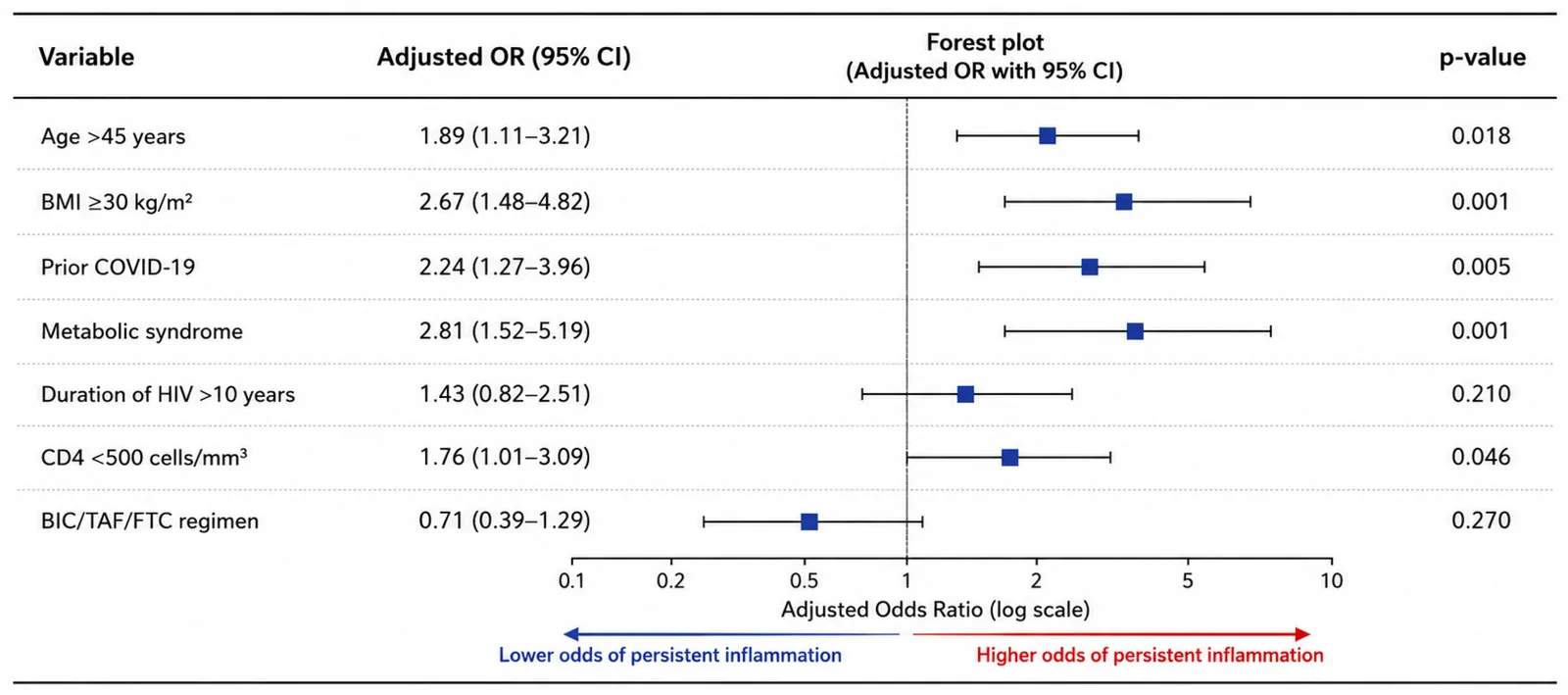
Multivariable logistic regression analysis of factors associated with persistent inflammation among virally suppressed people living with HIV (*n* = 231). Forest plot summarizing the adjusted odds ratios (ORs) and corresponding 95% confidence intervals (CIs) obtained from the multivariable logistic regression model. Each blue square represents the adjusted OR for the corresponding predictor, while the horizontal lines indicate the 95% CI. The vertical dashed line at OR = 1 represents the null value (no association). Variables with confidence intervals that do not cross 1 were considered independently associated with persistent inflammation. Age > 45 years, obesity (BMI ≥ 30 kg/m^2^), prior SARS-CoV-2 infection, metabolic syndrome, and CD4+ T-cell count < 500 cells/mm^3^ were independently associated with higher odds of persistent inflammation, whereas HIV infection duration > 10 years and treatment with a bictegravir/tenofovir alafenamide/emtricitabine (BIC/TAF/FTC)-based regimen were not significantly associated with the outcome. Odds ratios, 95% confidence intervals, and corresponding *p*-values are presented for each variable. Abbreviations: OR, odds ratio; CI, confidence interval; BMI, body mass index; HIV, human immunodeficiency virus; BIC/TAF/FTC, bictegravir/tenofovir alafenamide/emtricitabine.

A multivariable logistic regression analysis was subsequently performed to identify factors independently associated with persistent inflammation among virally suppressed patients (Table 4, Figure 3).

The model demonstrated an adequate goodness-of-fit according to the Hosmer–Lemeshow test (*p* = 0.48) and explained 31.0% of the variance in persistent inflammation (Nagelkerke R^2^ = 0.31). The model also showed acceptable discriminative ability, with an area under the receiver operating characteristic curve (AUC) of 0.79 (95% CI: 0.73–0.84).

As shown in Figure 2, age greater than 45 years was independently associated with an increased likelihood of persistent inflammation (OR = 1.89, 95% CI: 1.11–3.21, *p* = 0.018). Obesity (BMI ≥ 30 kg/m^2^) also emerged as a significant predictor (OR = 2.67, 95% CI: 1.48–4.82, *p* = 0.001).

A documented history of COVID-19 remained significantly associated with persistent inflammation after adjustment for potential confounders (OR = 2.24, 95% CI: 1.27–3.96, *p* = 0.005). Likewise, metabolic syndrome independently increased the odds of persistent inflammation (OR = 2.81, 95% CI: 1.52–5.19, *p* = 0.001).

Patients with CD4+ T-cell counts below 500 cells/mm^3^ were also more likely to exhibit persistent inflammation (OR = 1.76, 95% CI: 1.01–3.09, *p* = 0.046).

In contrast, HIV infection duration greater than 10 years and treatment with a BIC/TAF/FTC-based regimen were not independently associated with inflammatory status.

Taken together, these findings indicate that previous SARS-CoV-2 infection, metabolic abnormalities, obesity, and incomplete immune recovery represent the principal factors associated with persistent inflammation in this cohort.

## 4. Discussion

The present study provides further evidence that successful virological suppression does not necessarily translate into complete immunological recovery. Although all patients included in the secondary analysis had achieved sustained suppression of HIV replication, a substantial proportion exhibited elevated inflammatory biomarkers. More importantly, previous SARS-CoV-2 infection was independently associated with a less favorable inflammatory profile, even after adjustment for metabolic and HIV-related characteristics. Together, these findings support the concept that residual inflammation in treated HIV infection is associated with a combination of host-related factors and previous viral exposures rather than ongoing HIV replication alone [4,35,36].

Residual immune activation has become one of the central paradigms in contemporary HIV research. Previous studies have demonstrated that, despite effective antiretroviral therapy, biological processes such as persistent HIV reservoirs, microbial translocation secondary to impaired intestinal barrier dysfunction, and sustained activation of innate immune cells contribute to chronic inflammatory signaling [37,38,39,40]. These mechanisms are thought to maintain a low-grade inflammatory environment despite long-term virological control. However, the present study did not directly evaluate these biological pathways. Therefore, they are discussed here as established mechanisms described in the existing literature that may provide a biological framework for interpreting the observed associations, rather than as findings derived from our own analyses.

Within this context, one of the most striking findings of our study was the consistent association between previous SARS-CoV-2 infection and higher concentrations of CRP, IL-6, TNF-α, ESR, NLR, and PLR. Although the retrospective design precludes causal inference, the observed pattern is biologically plausible. Increasing evidence indicates that SARS-CoV-2 may leave behind a prolonged immunological imprint characterized by persistent cytokine production, endothelial activation, altered monocyte function, and sustained innate immune signaling that can persist for months after resolution of the acute infection [41,42,43,44]. Several of these pathways closely resemble those already implicated in chronic HIV infection [4,35,36], raising the possibility that the observed inflammatory profile reflects overlapping immune pathways related to both infections [35,36]. The present study did not assess these biological pathways directly; therefore, they should be considered potential explanatory mechanisms based on previous literature rather than findings derived from our analyses.

This interaction deserves particular attention because HIV and SARS-CoV-2 are known to affect several common immunological pathways. Previous studies have shown that chronic HIV infection is characterized by continuous immune stimulation driven by residual viral reservoirs, microbial translocation, and dysregulated cytokine production, whereas SARS-CoV-2 has been associated with prolonged activation of inflammatory cascades involving IL-6, TNF-α, interferon signaling, and activated monocyte populations [41,45,46]. Although these biological pathways were not directly evaluated in the present study, they provide a plausible framework for interpreting the observed association between prior SARS-CoV-2 infection and a less favorable inflammatory profile. Accordingly, our findings should be interpreted as observational associations rather than evidence that these mechanisms were active in our cohort or that they directly mediated the observed inflammatory changes. Nevertheless, the consistency of our findings with previous reports suggests that these mechanisms warrant further investigation in prospective mechanistic studies [41,45].

Another noteworthy observation is that the inflammatory profile associated with previous COVID-19 extended beyond conventional cytokine measurements. Patients with documented SARS-CoV-2 infection also demonstrated significantly higher NLR and PLR values, inexpensive hematological indices increasingly recognized as surrogate markers of chronic innate immune activation [47,48]. Although these indices lack the specificity of cytokine assays, they provide complementary information regarding systemic inflammatory status and have been associated with adverse clinical outcomes across several chronic inflammatory conditions, including HIV infection. Their consistent elevation in our cohort further supports an association between previous SARS-CoV-2 infection and multiple markers of systemic inflammation [47,49].

An equally important observation was the close relationship between residual inflammation and metabolic abnormalities. Patients classified as having persistent inflammation exhibited higher body mass index values and significantly higher rates of metabolic syndrome, dyslipidemia, and hepatic steatosis. These findings should not be interpreted as evidence that metabolic disturbances simply coexist with inflammation. Rather, they likely reflect the bidirectional relationship between chronic immune activation and metabolic dysfunction that has become increasingly recognized in treated HIV infection [45,50]. Sustained production of inflammatory cytokines, particularly IL-6 and TNF-α, contributes to insulin resistance, adipose tissue dysfunction, and alterations in lipid metabolism, whereas metabolically active adipose tissue itself further amplifies inflammatory signaling through continuous cytokine release [50,51]. Our findings therefore support the concept that metabolic abnormalities are both contributors to and consequences of chronic immune dysregulation.

Among these metabolic manifestations, hepatic steatosis deserves particular attention. Although liver-related outcomes were not the primary focus of the present study, steatosis was significantly more frequent among patients with previous SARS-CoV-2 infection and among those with persistent inflammation. Growing evidence indicates that metabolic dysfunction-associated steatotic liver disease in people living with HIV reflects a complex interaction between chronic inflammation, altered lipid metabolism, immune activation, and persistent metabolic stress rather than isolated hepatic injury [45,50,52]. It is therefore plausible that the higher prevalence of steatosis observed in our cohort represents another clinical manifestation of the chronic inflammatory environment accompanying long-term treated HIV infection.

Our study also demonstrated a consistent association between inflammatory activity and the degree of immune reconstitution. Patients with elevated inflammatory biomarkers had significantly lower CD4+ T-cell counts and lower CD4/CD8 ratios, while both CRP and IL-6 showed inverse correlations with CD4 recovery. Although this relationship has previously been reported, our findings reinforce its relevance in the post-COVID-19 era [3,53,54]. Residual inflammation may impair optimal immune restoration through continuous activation of both innate and adaptive immune compartments, promoting increased lymphocyte turnover, immune exhaustion, and incomplete recovery of T-cell homeostasis [53,54,55]. Conversely, suboptimal immune reconstitution may itself perpetuate inflammatory signaling by maintaining chronic immune dysregulation [54]. While the direction of this relationship cannot be established in a retrospective study, the coexistence of these findings highlights the close biological connection between inflammation and immune recovery in treated HIV infection.

The multivariable analysis further strengthened these observations. Even after adjustment for age, obesity, metabolic syndrome, HIV duration, immune status, and antiretroviral regimen, previous SARS-CoV-2 infection remained independently associated with residual inflammation. This finding suggests that previous SARS-CoV-2 infection was associated with an unfavorable inflammatory profile independently of conventional metabolic risk factors [41,56]. Rather than acting as an isolated trigger, previous SARS-CoV-2 infection may be associated with inflammatory pathways that overlap with those observed in treated HIV infection. However, our observational design does not allow determination of whether SARS-CoV-2 directly contributes to residual inflammation [55,56].

Interestingly, neither HIV duration nor treatment with a BIC/TAF/FTC-based regimen independently predicted inflammatory status. This observation is consistent with the evolving view that residual immune activation is determined predominantly by host-related biological factors rather than by the specific antiretroviral regimen once durable virological suppression has been achieved [55,57]. Contemporary ART effectively controls viral replication but does not eliminate residual viral reservoirs or fully restore immune homeostasis [55,56]. Consequently, therapeutic strategies directed exclusively toward viral suppression are unlikely to completely resolve residual inflammation, emphasizing the need for interventions targeting chronic inflammation and immune dysregulation in parallel with antiviral treatment [57].

Taken together, our findings reinforce the concept that residual inflammation represents one of the defining biological features of treated HIV infection during the post-COVID-19 era. Previous SARS-CoV-2 infection was independently associated with a less favorable inflammatory profile in this cohort, and this association may reflect biological pathways that overlap with the chronic inflammatory mechanisms already described in treated HIV infection. However, because of the retrospective observational design, our findings should be interpreted as evidence of association rather than proof that prior SARS-CoV-2 infection directly caused residual inflammation. Further mechanistic and longitudinal studies are needed to clarify the underlying biological pathways and the temporal relationship between prior SARS-CoV-2 infection and residual inflammation in people living with HIV despite successful antiretroviral therapy [57,58].

### 4.1. Strengths and Limitations

The present study has several important strengths. First, it was conducted in a well-characterized real-world cohort of people living with HIV receiving long-term suppressive antiretroviral therapy, allowing the evaluation of residual inflammation under conditions that closely reflect routine clinical practice. Unlike studies focusing primarily on virological outcomes, the present analysis simultaneously assessed inflammatory biomarkers, immune recovery, metabolic characteristics, and HIV-related clinical parameters, providing a comprehensive characterization of the inflammatory profile in treated HIV infection during the post-COVID-19 era.

Another important strength is the multimarker approach used to assess residual inflammation. By combining CRP, IL-6, TNF-α, ESR, NLR, and PLR, together with immunological parameters such as CD4+ T-cell count and CD4/CD8 ratio, the study captured complementary aspects of systemic inflammation and immune recovery rather than relying on a single biomarker. This integrated approach strengthens the characterization of the inflammatory phenotype associated with prior SARS-CoV-2 infection.

Several limitations should nevertheless be acknowledged. First, the retrospective observational design precludes causal inference and does not allow establishment of the temporal relationship between prior SARS-CoV-2 infection and persistent inflammation. Therefore, the observed findings should be interpreted as associations rather than evidence of causality. Second, this was a single-center study, which may limit the generalizability of the findings to other populations of people living with HIV.

Third, although only documented SARS-CoV-2 infections were considered, asymptomatic or unrecognized infections may have resulted in misclassification of some participants. In addition, important clinical information, including COVID-19 severity, viral variants, vaccination status, reinfections, and the exact interval between SARS-CoV-2 infection and biomarker assessment, was not consistently available and therefore could not be incorporated into the analyses. Vaccination and hybrid immunity may influence immune responses following SARS-CoV-2 infection in people living with HIV and therefore represent potential sources of residual confounding [59]. Importantly, the interval between documented SARS-CoV-2 infection and biomarker assessment was not consistently available. Consequently, we were unable to evaluate whether inflammatory biomarker levels varied according to the time elapsed since infection or to distinguish between post-acute inflammatory responses and longer-term residual inflammation.

Residual confounding should also be considered. Although the multivariable analysis adjusted for major metabolic and HIV-related factors, other clinically relevant variables known to influence inflammatory status, including smoking intensity, CD4 nadir, chronic viral hepatitis coinfection, previous malignancy, corticosteroid or other anti-inflammatory therapies, lifestyle characteristics, vaccination status, and other unmeasured comorbidities, were not consistently available in the medical records and therefore could not be included in the multivariable model. Consequently, their potential influence on the observed associations cannot be excluded. Previous studies have shown that vaccine-induced humoral and cellular immune responses may influence inflammatory and immunological profiles, further supporting the importance of vaccination status when interpreting inflammatory biomarkers [60].

Furthermore, the biological mechanisms underlying the observed associations could not be directly investigated because biomarkers of microbial translocation, intestinal barrier dysfunction, monocyte/macrophage activation, interferon signaling, and HIV reservoir size were not available in this retrospective cohort. In addition, the inflammatory classification was partly based on a cohort-specific IL-6 threshold using a cohort-specific IL-6 threshold, reflecting the absence of a universally accepted cutoff for residual inflammation in virally suppressed people living with HIV.

Finally, inflammatory biomarkers were measured at a single time point during routine follow-up, preventing assessment of their longitudinal evolution. Consequently, persistent inflammation was inferred from cross-sectional biomarker measurements rather than serial immunological assessments. Future prospective multicenter studies incorporating repeated biomarker measurements, detailed immune phenotyping, vaccination history, and characterization of COVID-19 severity will be essential to clarify the temporal evolution and biological mechanisms underlying the observed associations. Therefore, any discussion regarding these biological pathways should be interpreted as being based on previously published evidence rather than direct observations from the present study.

### 4.2. Future Directions

The findings of the present study raise several questions that warrant further investigation. Prospective longitudinal studies are needed to determine whether the inflammatory profile observed following SARS-CoV-2 infection represents a transient phenomenon or a sustained state of immune dysregulation in people living with HIV receiving suppressive antiretroviral therapy. Serial assessment of inflammatory biomarkers together with repeated immunological evaluations would provide a more comprehensive understanding of the temporal evolution of residual immune activation.

Future studies should also move beyond conventional inflammatory biomarkers and incorporate more detailed immune profiling. Simultaneous evaluation of cytokine networks, markers of monocyte and macrophage activation, microbial translocation, intestinal barrier dysfunction, and interferon signaling may help clarify the biological pathways linking previous SARS-CoV-2 infection with residual inflammation in treated HIV infection. Likewise, integrating measurements of HIV reservoir size and residual viral transcription may provide additional insights into the contribution of persistent viral antigens to chronic inflammatory signaling.

Another important area for future research involves identifying biological signatures capable of distinguishing patients at highest risk for long-term immune dysregulation. Combining inflammatory biomarkers with immune phenotyping, metabolic profiling, and virological parameters may facilitate the development of more precise risk stratification models and improve our understanding of the heterogeneity of immune recovery among virally suppressed individuals.

Future studies should also record the exact timing of SARS-CoV-2 infection in relation to biomarker assessment to better characterize the temporal evolution of inflammatory markers following COVID-19.

Finally, future investigations should explore whether residual inflammation following combined HIV and SARS-CoV-2 exposure is amenable to targeted therapeutic interventions. Better characterization of the molecular pathways sustaining chronic inflammation may contribute to the development of adjunctive immunomodulatory strategies aimed at restoring immune homeostasis, reducing chronic inflammatory burden, and ultimately improving long-term outcomes beyond virological suppression alone.

## 5. Conclusions

In this cohort of people living with HIV receiving suppressive antiretroviral therapy, prior SARS-CoV-2 infection was independently associated with a less favorable inflammatory profile, characterized by higher concentrations of CRP, IL-6, TNF-α, ESR, NLR, and PLR despite comparable virological control. These findings suggest that residual inflammatory activity may persist in some virally suppressed individuals despite effective HIV treatment.

Elevated inflammatory biomarker levels were also associated with less favorable immune reconstitution, as reflected by lower CD4+ T-cell counts and CD4/CD8 ratios, while prior SARS-CoV-2 infection, obesity, metabolic syndrome, and CD4+ T-cell counts below 500 cells/mm^3^ were independently associated with increased inflammatory activity. Together, these findings highlight the multifactorial nature of residual inflammation in treated HIV infection.

Because of the retrospective observational design, the present study demonstrates associations rather than causal relationships, and no conclusions regarding a direct causal effect of prior SARS-CoV-2 infection on residual inflammation can be drawn. Prospective longitudinal studies with repeated inflammatory biomarker measurements, detailed immune phenotyping, and comprehensive clinical characterization are needed to confirm these observations, better define the temporal relationship between prior SARS-CoV-2 infection and persistent inflammation, and further elucidate the underlying biological mechanisms.

## Figures and Tables

**Table 1:** Baseline characteristics of HIV-positive patients according to SARS-CoV-2 infection history.

Parameter	Total Cohort *n* = 286	Prior COVID-19 *n* = 132	No Documented COVID-19 *n* = 154	Effect Size (95% CI)	*p*-Value
Age, years	45.8 ± 11.6	46.2 ± 10.9	45.4 ± 12.1	Cohen’s d = 0.07	0.56
Male sex, *n* (%)	178 (62.2)	84 (63.6)	94 (61.0)	OR = 1.12 (95% CI: 0.69–1.81)	0.65
BMI, kg/m^2^	26.7 ± 4.8	27.4 ± 5.1	26.1 ± 4.5	Cohen’s d = 0.27	0.024
Current smoking, *n* (%)	96 (33.6)	48 (36.4)	48 (31.2)	OR = 1.26 (95% CI: 0.77–2.06)	0.35
Duration of HIV infection, years	9.4 ± 5.7	9.7 ± 5.9	9.1 ± 5.5	Cohen’s d = 0.11	0.38
Duration of ART, years	8.1 ± 5.2	8.4 ± 5.4	7.8 ± 5.0	Cohen’s d = 0.12	0.33
Viral suppression < 50 copies/mL, *n* (%)	231 (80.8)	109 (82.6)	122 (79.2)	OR = 1.24 (95% CI: 0.69–2.25)	0.47
CD4 count, cells/mm^3^	612 ± 238	598 ± 229	624 ± 246	Cohen’s d = −0.11	0.36
CD4/CD8 ratio	0.74 ± 0.31	0.71 ± 0.29	0.77 ± 0.32	Cohen’s d = −0.20	0.09
Metabolic syndrome, *n* (%)	82 (28.7)	46 (34.8)	36 (23.4)	OR = 1.75 (95% CI: 1.05–2.94)	0.033
Dyslipidemia, *n* (%)	116 (40.6)	61 (46.2)	55 (35.7)	OR = 1.55 (95% CI: 0.96–2.49)	0.071
Hepatic steatosis, *n* (%)	74 (25.9)	42 (31.8)	32 (20.8)	OR = 1.78 (95% CI: 1.04–3.04)	0.034
BIC/TAF/FTC regimen, *n* (%)	126 (44.1)	59 (44.7)	67 (43.5)	OR = 1.05 (95% CI: 0.66–1.68)	0.84
DTG-based regimen, *n* (%)	88 (30.8)	42 (31.8)	46 (29.9)	OR = 1.10 (95% CI: 0.66–1.81)	0.72
PI-based regimen, *n* (%)	42 (14.7)	21 (15.9)	21 (13.6)	OR = 1.20 (95% CI: 0.62–2.31)	0.58

Continuous variables are presented as mean ± standard deviation (SD), whereas categorical variables are presented as number (percentage). Effect sizes are reported as Cohen’s d for continuous variables and odds ratios (ORs) with corresponding 95% confidence intervals (CIs) for categorical variables. Comparisons between groups were performed using the independent samples *t*-test for continuous variables and the chi-square test or Fisher’s exact test, as appropriate. Abbreviations: ART, antiretroviral therapy; BMI, body mass index; OR, odds ratio; CI, confidence interval; CD4, cluster of differentiation 4; BIC/TAF/FTC, bictegravir/tenofovir alafenamide/emtricitabine; DTG, dolutegravir; PI, protease inhibitor.

**Table 2:** Inflammatory biomarkers according to SARS-CoV-2 infection history.

Parameter	Prior COVID-19 (*n* = 132)	No Documented COVID-19 (*n* = 154)	Mean Difference (95% CI)	*p*-Value
CRP (mg/L)	5.8 ± 3.4	3.9 ± 2.7	1.90 (1.18 to 2.62)	<0.001
IL-6 (pg/mL)	7.4 ± 3.1	5.1 ± 2.6	2.30 (1.63 to 2.97)	<0.001
TNF-α (pg/mL)	12.6 ± 4.8	9.8 ± 3.9	2.80 (1.77 to 3.83)	<0.001
ESR (mm/h)	21.3 ± 10.4	16.8 ± 8.7	4.50 (2.24 to 6.76)	0.001
Leukocyte count (×10^9^/L)	6.8 ± 1.9	6.5 ± 1.8	0.30 (−0.13 to 0.73)	0.19
Neutrophils (%)	61.2 ± 10.8	57.3 ± 9.9	3.90 (1.47 to 6.33)	0.003
Lymphocytes (%)	29.4 ± 8.5	32.8 ± 8.2	−3.40 (−5.35 to −1.45)	0.002
NLR	2.44 ± 1.13	1.92 ± 0.89	0.52 (0.28 to 0.76)	<0.001
PLR	152.6 ± 51.2	136.4 ± 45.7	16.20 (4.81 to 27.59)	0.007

Continuous variables are presented as mean ± standard deviation (SD), together with the mean difference and corresponding 95% confidence interval (CI). Comparisons between patients with and without documented prior SARS-CoV-2 infection were performed using the independent samples *t*-test. Positive mean differences indicate higher values in the prior COVID-19 group. Abbreviations: CRP, C-reactive protein; IL-6, interleukin-6; TNF-α, tumor necrosis factor-alpha; ESR, erythrocyte sedimentation rate; NLR, neutrophil-to-lymphocyte ratio; PLR, platelet-to-lymphocyte ratio; CI, confidence interval.

**Table 3:** Clinical outcomes according to inflammatory status among virally suppressed patients.

Parameter	Low Inflammation (*n* = 115)	High Inflammation (*n* = 116)	Mean Difference (95% CI)	*p*-Value
Age (years)	43.1 ± 10.5	48.7 ± 11.4	5.60 (2.76 to 8.44)	<0.001
BMI (kg/m^2^)	25.4 ± 4.3	28.6 ± 5.1	3.20 (1.98 to 4.42)	<0.001
Metabolic syndrome, *n* (%)	22 (19.1)	51 (44.0)	OR 3.34 (95% CI: 1.82–6.14)	<0.001
Dyslipidemia, *n* (%)	34 (29.6)	59 (50.9)	OR 2.46 (95% CI: 1.40–4.31)	0.001
Hepatic steatosis, *n* (%)	18 (15.7)	42 (36.2)	OR 3.05 (95% CI: 1.60–5.83)	<0.001
CD4 count (cells/mm^3^)	648 ± 231	584 ± 218	−64 (−122 to −6)	0.031
CD4/CD8 ratio	0.81 ± 0.28	0.69 ± 0.25	−0.12 (−0.19 to −0.05)	0.004

Patients were classified as having either low inflammatory burden or persistent inflammation according to the predefined study criteria. Continuous variables are presented as mean ± standard deviation (SD), together with the mean difference and corresponding 95% confidence interval (CI). Categorical variables are presented as number (percentage), together with odds ratios (ORs) and 95% confidence intervals. Comparisons between groups were performed using the independent samples *t*-test for continuous variables and the chi-square test or Fisher’s exact test, as appropriate. Abbreviations: BMI, body mass index; OR, odds ratio; CI, confidence interval; CD4, cluster of differentiation 4.

**Table 4:** Multivariable logistic regression analysis for persistent inflammation among virally suppressed patients.

Variable	OR	95% CI	*p*-Value
Age > 45 years	1.89	1.11–3.21	0.018
BMI ≥ 30 kg/m^2^	2.67	1.48–4.82	0.001
Prior COVID-19	2.24	1.27–3.96	0.005
Metabolic syndrome	2.81	1.52–5.19	0.001
Duration of HIV > 10 years	1.43	0.82–2.51	0.21
CD4 < 500 cells/mm^3^	1.76	1.01–3.09	0.046
BIC/TAF/FTC regimen	0.71	0.39–1.29	0.27

Adjusted odds ratios (ORs), corresponding 95% confidence intervals (CIs), and *p*-values are presented for all variables included in the multivariable model. An OR >1 indicates higher odds of persistent inflammation, whereas an OR <1 indicates lower odds of persistent inflammation after adjustment for the other variables included in the model. Variables with *p* < 0.05 were considered independently associated with the outcome. Abbreviations: OR, odds ratio; CI, confidence interval; BMI, body mass index; CD4, cluster of differentiation 4; HIV, human immunodeficiency virus; COVID-19, coronavirus disease 2019; BIC/TAF/FTC, bictegravir/tenofovir alafenamide/emtricitabine.

## Data Availability

The original contributions presented in this study are included in the article. Further inquiries can be directed to the corresponding authors.
